# The risk of heart failure associated with the use of noninsulin blood glucose-lowering drugs: systematic review and meta-analysis of published observational studies

**DOI:** 10.1186/1471-2261-14-129

**Published:** 2014-09-26

**Authors:** Cristina Varas-Lorenzo, Andrea V Margulis, Manel Pladevall, Nuria Riera-Guardia, Brian Calingaert, Lorna Hazell, Silvana Romio, Susana Perez-Gutthann

**Affiliations:** RTI Health Solutions, Trav. Gracia 56 Atico 1, 08006 Barcelona, Spain; RTI Health Solutions, 200 Park Offices Drive, Research Triangle Park, NC 27709 USA; Drug Safety Research Unit, Bursledon Hall, Blundell Lane, Southampton, S031 1AA UK; Department of Medical Informatics, Erasmus University Medical Center, Rotterdam, Netherlands; Department of Statistics and Quantitative Methods, Division of Biostatistics, Epidemiology and Public Health, Laboratory of Healthcare Research and Pharmacoepidemiology, University Milano-Bicocca, Milan, Italy

**Keywords:** Blood glucose-lowering drugs, Type 2 diabetes mellitus, Heart failure risk, Meta-analysis, Cardiovascular safety, Pharmacoepidemiology, Observational studies

## Abstract

**Background:**

Patients with type 2 diabetes mellitus (T2DM) are at high risk of heart failure. A summary of the effects of blood glucose-lowering drugs other than glitazones on the risk of heart failure in routine clinical practice is lacking. The objective of this study was to conduct a systematic review and meta-analysis of observational studies on the risk of heart failure when using blood glucose-lowering drugs.

**Methods:**

We systematically identified and reviewed cohort and case–control studies in which the main exposure of interest was noninsulin blood glucose-lowering medications in patients with T2DM. We searched Medline, Embase, and the Cochrane Library to identify publications meeting prespecified eligibility criteria. The quality of included studies was assessed with the Newcastle-Ottawa Scale and the RTI item bank. Results were combined using fixed and random-effects models when at least 3 independent data points were available for a drug-drug comparison.

**Results:**

The summary relative risk of heart failure in rosiglitazone users versus pioglitazone users (95% CI) was 1.16 (1.05-1.28) (5 cohort studies). Heterogeneity was present (*I*^*2*^ = 66%). For new users (n = 4) the summary relative risk was 1.21 (1.14-1.30) and the heterogeneity was reduced (*I*^*2*^ = 31%);. The summary relative risk for rosiglitazone versus metformin was 1.36 (95% CI, 1.17-1.59) (n = 3). The summary relative risk (95% CI) of heart failure in sulfonylureas users versus metformin users was 1.17 (95% CI, 1.06-1.29) (5 cohort studies; *I*^*2*^ = 24%) and 1.22 (1.02-1.46) when restricted to new users (2 studies).

Information on other comparisons was very scarce. Information on dose and duration of treatment effects was lacking for most comparisons. Few studies accounted for disease severity; therefore, confounding by indication might be present in the majority of the within-study comparisons of this meta-analysis.

**Conclusions:**

Use of glitazones and sulfonylureas was associated with an increased risk of heart failure compared with metformin use. However, indication bias cannot be ruled out. Ongoing large multidatabase studies will help to evaluate the risk of heart failure in treated patients with diabetes, including those using newer blood glucose-lowering therapies.

**Electronic supplementary material:**

The online version of this article (doi:10.1186/1471-2261-14-129) contains supplementary material, which is available to authorized users.

## Background

Progressive loss of the insulin secretory capacity of the pancreatic beta cells and insulin resistance are the two mechanisms involved in the pathophysiology of type 2 diabetes mellitus (T2DM) [1]. Earlier medications for the treatment of T2DM were targeted to stimulate or replace endogenous insulin secretion. Newly developed agents are indirectly or directly targeted to treat insulin resistance, which usually precedes the clinical manifestations of diabetes. Insulin resistance, a state in which a given concentration of insulin is associated with a subnormal glucose response [2], and diabetes are both associated with an increased risk of cardiovascular disease and are often accompanied by a constellation of other cardiovascular risk factors. In fact, T2DM has been considered as equivalent to coronary heart disease for cardiovascular risk prediction and prevention [3, 4]. Therefore, newer medications are intended not only to control hyperglycemia and reduce the risk of microvascular complications but also to reduce the risk of macrovascular complications [5]. However, as it has been also pointed out by others, “therapies for diabetes mellitus have not traditionally been rigorously evaluated for the risk of developing incident or worsening heart failure” [6].

The glitazones, with a predominant mechanism of action on peroxisome proliferator-activated receptor-γ genes, were considered as potentially having additional clinical benefit due to their insulin-sensitizing effect. Fluid retention was identified among the safety concerns before approval, but other cardiovascular safety concerns arose after their availability in 2000. The European Medicines Agency (EMA) suspended the authorization of rosiglitazone in September 2010 due to concerns about the associated increased risk of acute coronary syndrome, while the United States (USA) Food and Drug Administration (FDA) imposed a restricted prescription program in November 2011 [7]. Two years later, the FDA lifted its earlier restrictions after reviewing the results of the 2009 RECORD clinical trial, which did not show any increase in the risk of acute myocardial infarction associated with the drug [8].

Patients with T2DM are at high risk of heart failure due to the coexistence of diabetes with other risk factors such as hypertension, ischemic heart disease, and kidney function decline and because the diabetic myocardium might be more sensitive to the deleterious effect of cardiovascular risk factors [9]. Diabetes is a strong predictor of incident heart failure [10]. Almost 40% of patients hospitalized for acute decompensated heart failure have diabetes [11]. In the context of T2DM, once heart failure is present, the patient’s prognosis is poor [12]. The risk of heart failure associated with blood glucose-lowering medications may arise as an undesired effect of the drugs or as a consequence of their lack of effectiveness. For example, glitazones are associated with weight gain and fluid retention leading to edema, which might trigger the development of congestive heart failure in predisposed patients. Poor glycemic control in patients with T2DM is also associated with increased risk of heart failure [13, 14].

The 2012 joint clinical guidelines of the American Diabetes Association (ADA) and the European Association for the Study of Diabetes (EASD) emphasized that the choice of blood glucose-lowering agent should focus on drug safety, especially protecting against hypoglycemia, heart failure, renal dysfunction, bone fractures, and drug-drug interactions [15]. However, clinical guidelines have changed over time. Metformin and rosiglitazone were both initially contraindicated in patients with heart failure. In the case of metformin, this contraindication was due to the potentially increased risk for lactic acidosis, which was why the related drugs phenformin and buformin were removed from the market. At present, metformin is being recommended as first-line therapy in clinically stable patients with heart failure if their ventricular dysfunction is not severe. Glitazones are contraindicated in patients with heart failure due to an increased risk of exacerbations [16–19]. Therefore, most of the available systematic reviews focused either on patients with diabetes already diagnosed with heart failure and compared metformin with other T2DM treatments [20–22] or on the risk of heart failure associated with rosiglitazone compared with pioglitazone [23, 24]. Another systematic review evaluated the risk of heart failure associated with other drug comparisons, but did not numerically summarize the findings [25]. The effects of blood glucose-lowering drugs on the risk of heart failure in routine clinical practice, other than the risk observed for glitazones, has not been systematically reviewed and integrated.

The present research effort is part of the Safety Evaluation of Adverse Reactions in Diabetes (SAFEGUARD) project (http://www.safeguard-diabetes.org/). The goal of SAFEGUARD is to evaluate the cardiovascular and pancreatic safety of oral blood glucose-lowering medications in patients with T2DM. For this purpose, the project includes an ongoing observational study of aggregated data from more than 1.7 million patients in Europe and the USA, the systematic review of available evidence from published observational studies and clinical trials, and the implementation of state-of-the-art mechanistic studies. Systematic reviews were performed separately for observational studies and clinical trials as recommended by the *Cochrane Handbook for Systematic Reviews of Interventions*[26]. In this report, we summarize the results of a systematic review of published observational studies on the risk of heart failure to address the above-mentioned gaps.

## Methods

The methods used to define the specifications for search, selection, abstraction, quality assessment, data synthesis, and analysis of the published literature are described below.

### Literature search

We conducted a systematic literature search in Medline, Embase, and the Cochrane Library with medical subject headings (MeSH) terms and free-text words for noninsulin blood glucose-lowering drugs, heart failure (the focus of this research), and other cardiovascular outcomes (acute coronary syndrome or acute myocardial infarction, stroke, cardiovascular death, ventricular arrhythmia, and sudden cardiac death), which are being evaluated in a separately review. The search was conducted on November 11, 2011, and was limited to observational studies on humans, including systematic reviews, meta-analyses, and original articles, with no publication date or language restrictions (Medline search terms are included in the Additional file 1). Reference lists of systematic reviews and meta-analyses identified during the study selection process were examined for eligible studies not previously identified. We conducted an updated literature search in September 2014, and the impact of the fewer additional studies that were identified is reported in the Discussion section.

### Study selection and data abstraction

Studies were eligible if they were cohort or case–control studies on individuals with T2DM and provided relative risk estimates of heart failure comparing two or more noninsulin blood glucose-lowering drugs, in monotherapy or combination therapy, individually or combined in drug classes. Relative risk estimates had to be adjusted, at a minimum, for age and sex. If relative risk estimates were not provided but data were presented to enable estimation of age-and-sex-adjusted relative risks, the study was considered eligible. No exclusion criteria were implemented.

Titles and abstracts were reviewed for study eligibility by at least two researchers. Disagreements were solved by consensus with a third researcher. Selected studies underwent full-text review for final selection decisions. Data were abstracted by one researcher using standardized data collection forms in Microsoft Access and Excel designed a priori for the present study and adapted after a pilot test. A second researcher reviewed the extracted data for accuracy and completeness. Disagreements were solved by consensus.

When results of any given comparison were available from more than one study on largely overlapping study populations, we selected the most recent study. Within each study, when multiple results were available, we selected those with the longest observation period and the most extensive covariate adjustment. Results comparing drugs of interest and insulin were retained as long as the study population consisted of individuals with T2DM, but results comparing drugs of interest to untreated subjects were not abstracted. When the data to extract from an article appeared only in figures in the original study, or for clarifying information for study inclusion decisions, authors of a few studies were contacted with requests for point estimates and confidence intervals.

### Quality assessment

We assessed the quality of each study included in the systematic review using two tools that investigate the risk of bias in several domains, the Newcastle-Ottawa Quality Assessment Scale (NOS) [27] and the RTI item bank on risk of bias and precision [28, 29]. (See Additional file 1 for a detailed description of the two instruments). We tailored both tools to the topic of this systematic review. The tools were applied to each article separately by two researchers (MP, NR, or AVM); disagreements were solved by consensus. Findings of high and unclear risk of bias for each individual study based on the RTI item bank are displayed in graphs in Additional file 1.

### Data synthesis and analysis

Quantitative analysis was conducted using Review Manager (RevMan) software version 5.2.3 (The Nordic Cochrane Centre, Copenhagen). For each comparison with at least three independent point estimates available, we estimated summary relative risks and 95% confidence intervals (CIs) for heart failure using both random and fixed-effects models [30]. We inverted some reported relative risks within the same comparison group to use a common reference drug; for example, converting the comparison of pioglitazone versus rosiglitazone into rosiglitazone versus pioglitazone.

We generated forest plots that included adjusted relative risks and weights for pooled analysis for individual studies, as well as the effect size estimate (95% CI) for the aggregated results under the random effects model. If within a given study and for a given comparison, results were reported separately for monotherapy and combination therapy, we estimated the summary relative risk using fixed-effects models for inclusion in the overall forest plot. If the groups did not include independent subjects, the group with the most precise relative risk was included in the forest plot. Where statistical meta-analysis was not appropriate due to insufficient data, relative risk estimates from each study were reported separately. In the overall forest plot, for completeness, we provided the summary relative risk estimate based on only two studies for some comparisons. Between-study heterogeneity was assessed by graphical inspection of forest plots and with Cochran’s *χ*^*2*^ test of homogeneity. *Tau*^*2*^ is also presented as an estimate of the between-study variance when there was statistical evidence of heterogeneity (*P* value < 0.10) according to the *χ*^*2*^ test from the random-effects models. The Higgins *I*^*2*^ statistic was used to describe the percentage of between-study variability estimates of the total variability that is attributable to true heterogeneity rather than chance; a threshold of 50% was considered indicative of substantial to considerable heterogeneity [31].

To explore sources of heterogeneity and better understand the evidence, we implemented subgroup analyses designed a priori. When possible, we stratified according to study design, regimen (monotherapy only and combined therapy), type of drug use (new users and all users including prevalent users), type of event (incident heart failure only and combined incident plus prevalent heart failure), and finally the age range of the source population. The *χ*^*2*^ test was used to test for homogeneity between subgroups. When stratification was not possible due to the number of studies, we restricted the analysis to one of the study subgroups (i.e., new users only).

Publication bias was examined by visual evaluation of funnel plots.

The present report follows MOOSE (Meta-analysis Of Observational Studies in Epidemiology) guideline/checklist [32], which is included in the Additional file 1. The present systematic review of published observational studies does not require ethics approval.

## Results

### Study selection and characteristics of included studies

**Figure 1:**
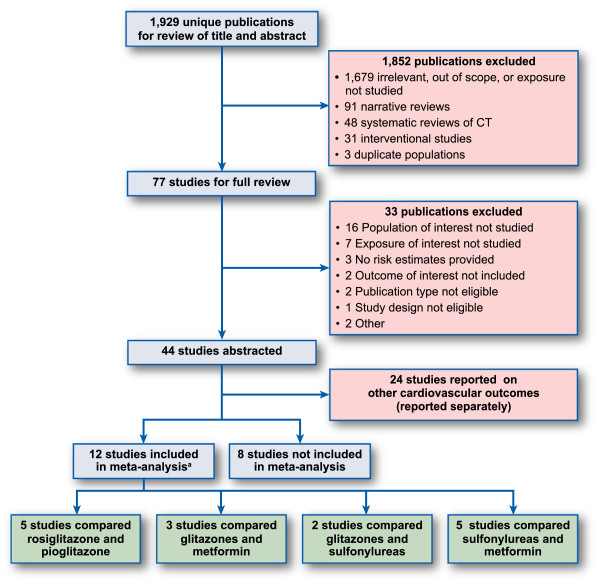
**Flow Diagram of Study Identification and Selection Process.** Note: No study was added by cross-referencing. ^a^Some studies contributed to more than one drug-drug comparison.

Figure 1 displays the study selection process. From the 44 studies selected for the systematic review of cardiovascular outcomes, we identified 20 studies evaluating the risk of heart failure [33–52] (see Table 1). Of these, 12 contributed to the meta-analysis, with six drug comparisons: rosiglitazone versus pioglitazone (5 studies) [33, 34, 37, 43, 44], rosiglitazone versus metformin (3 studies) [36, 40, 42], pioglitazone versus metformin (2 studies) [36, 42], rosiglitazone versus sulfonylureas (1 study) [35], pioglitazone versus sulfonylureas (2 studies) [36, 38], and sulfonylureas versus metformin (5 studies) [35, 38, 39, 41, 42]. The other 8 studies could not be included in the meta-analysis due to the heterogeneity of the comparisons, which often involved reference groups combining several medications. One study was performed in patients with diagnosed heart failure who were followed for hospital readmission for heart failure or death [49].

**Table 1:** **Main features of studies included in the systematic literature review**

Author, year	Source population, study period	Study design, population, age	Diabetes Type 2 population definition	Study endpoint ascertainment (Number of cases)	Case validation	Exposure assessment	Exposure recency	Exposure Group(s) (n) vs. reference group (n)
								A: Comparison(s) Contributing to Meta-analysis
								B: Other Reported Comparison(s)
Studies included in the meta-analysis (n = 12)								
Chou [33]	Taiwan Longitudinal Health Insurance Database 1998-2006	Cohort N = 7725 < 110 years	ICD-9 code 250.xx in the study period with prescriptions for glitazones	Incident outpatient and emergency department diagnoses of nonfatal HF (ICD-9: 428 and diuretic use) (N = 356)	None	Prevalent and new users Dispensed prescriptions	Current, continuous use of more than 120 days in last 180 days after index date of cohort inclusion	A: Rosiglitazone (n = 6048) vs. pioglitazone (n = 1677); as add-on treatment to other medications
Graham [34]	Medicare, USA 2006-2009	Cohort N = 227571 ≥ 65 years	First prescriptions for glitazones	Hospitalization for HF (ICD-9: 402.x1; 404.x3; 428) (N = 3307)	External; PPV: range, 85%-96%	New users Dispensed prescriptions	Current, continuous use including 7 days gap	A: Rosiglitazone (n = 67593) vs. pioglitazone (n = 159978)
Horsdal [35]	Danish National Registries, Denmark 1996-2004	Cohort N = 8494 Patients hospitalized for AMI receiving monotherapy with OHA	Subjects were classified as with T1DM and excluded if they were younger than 30 years at the time of their first related prescription or diagnosis and had never received a prescription for an oral glucose-lowering drug. Subjects with T2DM were those with codes for diabetes mellitus who had not received pharmacotherapy, or had received prescriptions for oral glucose-lowering drugs, or were older than 30 years when they had their first diagnostic code or prescription.	Hospital admission for HF (ICD-10: I11.0, I13.0, I13.2, I25.5, I42.0, I42.7, I42.8, I42.9, I50.0, I50.1, I50.9) within 1 year of AMI (N = NR)	None	Prevalent and new users Dispensed prescriptions	At least one prescription of study drug within 90 days before hospitalization	A: Metformin monotherapy (n = 396) vs. SU monotherapy (n = 2382)
Hsiao [36]	Taiwan Longitudinal Health Insurance Database 2001-2005	Cohort N = 473483 Age, NR	Subjects with their first ambulatory visit with ICD-9-CM code 250.xx who were prescribed oral blood glucose lowering agents at least three times. Subjects were excluded if they had T1DM (ICD-9-CM codes 250.x1) or if they had been prescribed insulin only during the study period.	Hospitalization for HF (ICD-9: 428, 402.01, 402.11, 402.91; 404) (N = 2530)	None	New users Dispensed prescriptions	Current, continuous use during study period	A: Pioglitazone monotherapy (n = 495) or rosiglitazone monotherapy (n = 2093) vs. metformin-based therapy (n = 46444) and vs. SU-based therapy (n = 97651) B: Pioglitazone + SU + metformin (n = 9510) vs. Rosiglitazone + SU + metformin (n = 39962) Pioglitazone + metformin (n = 774) vs. Rosiglitazone + metformin (n = 2408) Pioglitazone + SU (n = 1231) vs. Rosiglitazone + SU (n = 5141)
Juurlink [37]	Ontario diabetes database, Canada 2002-2008	Cohort N = 39736 ≥ 66 years	First prescription for a glitazone.	Hospitalization for HF (ICD-10: I50) (N = 1330)	External; PPV ≈ 90%	New users Dispensed prescriptions	Current use, if refill occurred < 1.5 times the days’ supply of the preceding glitazone claim	A: Pioglitazone (n = 16951) vs. rosiglitazone (n = 22785)
Karter, [38]	Kaiser Permanente, diabetes registry, USA 1999-2001	Cohort N = 23440 Age, mean (SD): 58.9 (12.3) years	Diagnosis of T2DM in the Kaiser Permanente Northern California Diabetes Registry, initiation of diabetes treatment, and at least one refill of the initial drug.	Incident; excluded within 5 years prior to baseline outpatient, emergency or hospital discharge diagnoses of CHF Hospitalization for CHF (ICD-9: 428; 401.91, 402.01, 402.11, 402.91; 404.01, 404.03, 404.11, 404.13, 404.93, 425.1, 425.4, 425.5, 425.7) (N = 320)	External, PPV = 97%	New users Dispensed prescriptions	Current, continuous use during study period	A: Pioglitazone (n = 3556) or metformin (n = 11937) vs. SU (n = 5921) as single index therapy but with other maintenance therapy
Koro [39]	GPRD, United Kingdom 1987-2001	Nested case–control N = 9089 ≥ 30 years	The cohort follow-up started with the earliest diagnosis of T2DM in the electronic medical record.	First ever diagnosis of CHF according to GPs recorded OXMIS/Read codes (N = 1301)	External	Prevalent and new users Prescriptions issued	Current use in last 3 months before index date (case date or matched date for controls)	A: Metformin (152 cases; 915 controls) or metformin + SU (177 cases, 817 controls) vs. SU (591 cases, 3547 controls)
Loebstein [40]	Maccabi Healthcare Services, Israel 2000-2007	Cohort N = 15436 Age, mean (SD): 59.1 (11.4) years	Subjects in the Maccabi diabetes registry with prescriptions for rosiglitazone or metformin for at least 6 months.	Hospitalization for HF (wrong code reported as ICD-9 150) (N = NR)	None	Prevalent and new users Dispensed prescriptions	Current, continuous use within study period with gaps not longer than 3 months	A: Rosiglitazone monotherapy (n = 745) or in combination with metformin (n = 2753) vs. metformin monotherapy (n = 11938) (Formulary restriction for rosiglitazone only if not adequate control after SU, metformin, or both)
McAlister [41]	Saskatchewan Health beneficiaries, Canada 1991-1996	Cohort N = 5631 ≥ 30 years	New prescription for an oral blood glucose-lowering drug. The authors describe the study population as subjects with recent onset of diabetes.	Incident during prior 3 years Hospitalization for CHF or physician visit with HF diagnosis (ICD-9: 428) (N = 981)	External	New users Dispensed prescriptions	At least one prescription for an OHA	A: SU (glyburide, chlorpropamide or tolbutamide) monotherapy (n = 4162) vs. metformin monotherapy (n = 1469)
Tzoulaki, [42]	GPRD, United Kingdom 1990-2005	Cohort N = 91521 35-90 years	One episode of care associated with a clinical or referral event for diabetes and prescriptions for oral blood glucose-lowering treatment.	First ever diagnosis of CHF according to Read codes (N = 6900)	External; confirmed 83% of the CHF diagnoses	Prevalent and new users Prescriptions issued	Current, continuous intervals of use within the study period	A: First-generation SU monotherapy (n = 6053) or second-generation SU monotherapy (n = 58095) or rosiglitazone monotherapy (n = 8442) and combination therapy (n = 9640) or pioglitazone including monotherapy and combination therapy (n = 3816) vs. metformin (n = 68181) B: Glibenclamide or gliclazide or glimepiride or glipizide or gliquidone vs. metformin (n = 68181)
Wertz [43]	HealthCore Integrated Research Database, USA 2001-2005	Cohort N = 36628 ≥ 18 years	First prescription for glitazones.	Hospitalizations for AHF (ED visits included) (ICD-9: 402.01, 402.11, 402.91; 404.01, 404.03, 404.11, 404.13, 404.91, 404.93) (N = 508)	None	New users Dispensed prescriptions	Current use, if refill occurred < 1.5 times the days’ supply of the preceding claim for TZD	A: Rosiglitazone (n = 14469) vs. pioglitazone (n = 14469)
Winkelmayer [44]	Medicare, New Jersey, USA 1999-2005	Cohort N = 28361 > 65 years	First prescription for a glitazone, regardless of previous treatment with other diabetes drug.	Hospitalization for CHF (ICD-9: 428) (N = 1259)	External PPV = 94%	New users Dispensed prescriptions	Current, continuous use until 60 days after the supply date of their most recently filled prescription duration or until switching to other TZD	A: Rosiglitazone (n = 14101) vs. pioglitazone (n = 14260)
**Studies reviewed but not included in the meta-analysis (n = 8)**								
Delea [45]	Pharmetrics integrated outcomes database USA 1997-2001	Cohort N = 33544 ≥ 18 years	Subjects with one or more claims with ICD-9 codes 250.x0 or 250.x2 and one or more prescriptions for oral blood glucose-lowering drugs (first prescription in the case of glitazones).	First ever inpatient or outpatient claim for CHF (ICD-9-CM: 402.11, 402.91, 428, 428.0, 428.1, 428.9) (N = 423)	None	New users Dispensed prescriptions	Current, continuous use with permitted gaps of 90 days after the last refill	A: NA B: Troglitazone or rosiglitazone or pioglitazone (n = 5441) vs. other OHA or vs. non-TZD noninsulin OHA or vs. no use of TZD (n = 28103)
Habib [46]	Henry Ford, USA 2000-2006	Cohort N = 19171 > 18 years	Subjects with one or more claims with ICD-9 code 250.xx and one or more prescriptions for oral blood glucose-lowering drugs.	Hospitalization for CHF (codes not reported) (N = 2725) All-cause mortality	None	Prevalent and new users Dispensed prescriptions	Days’ supply of medication dispensed in a 6-month period divided by the number of days	A: NA B: Rosiglitazone, pioglitazone, or rosiglitazone + pioglitazone(n = 4580) vs. other OHA or vs. nonuse of TZD (n = 14591)
Hartung, [47]	Medicaid USA 1999-2001	Case–control N = 1940 ≥ 18 years	Subjects were eligible as cases or controls if they had one or more records with ICD-9 code 250.xx as primary diagnosis and one or more prescriptions for oral blood glucose-lowering drugs.	Hospitalization for HF (DRG 127.xx) (N = 288) (Controls: hospitalizations for other conditions)	None	Prevalent and new users Dispensed prescriptions	Current, at least use of one prescription within 60 days before index hospitalization for cases and controls	A: NA B: TZD (n = 275) vs. nonuse of TZD (n = 1665) B: TZD (n = 275) vs. nonuse of TZD (n = 1665)
Horsdal [48]	Danish National Registries, Denmark 1996-2004	Cohort N = 3930 Patients aged ≥ 30 years hospitalized for AMI	At least one prescription for a sulfonylurea in the 90 days before hospitalization for myocardial infarction.	Hospital admission for HF within 1 year of AMI (ICD 10: I11.0, I13.0, I13.2, I25.5, I42.0, I42.6-I42.9, I50.0, I50.1, I50.9) (N = 329)	External	Prevalent and new users Dispensed prescriptions	Use of at least one prescription of study drug within 90 days before the index hospitalization for AMI	A: NA B: Gliclazide (n = 216) or glimepiride (n = 906) or glipizide (n = 616) or glibenclamide (n = 1238) vs. tolbutamide (n = 472)
Hsiao [49]	Taiwan Longitudinal Health Insurance 2001-2005	Cohort N = 8139 Patients hospitalized for CHF and prescribed either TZD or SU monotherapy	At least one code for T2DM (ICD-9 code 250.xx [sic]). Subjects were excluded if they had T1DM (mechanism for identification not explained) or if they only had prescriptions for insulin during the study period (description not clear).	Hospital readmission for HF (ICD-9: 428, 402.01, 402.11, 402.91, 404.01, 404.03, 404.11, 404.13, 404.91, 404.92) (N = 2536)	None	Prevalent and new users Dispensed prescriptions	Cumulative use (DDD) since index hospitalization	A: NA B: TZD (n = 7023) vs. SU (n = 204)
Lipscombe [50]	Ontario Health Care Database, Canada 2002-2006	Nested case–control N = 159026 ≥ 66 years	Subjects registered in the Ontario Diabetes Database were followed since their last prescription for an oral hypoglycemic agent.	Hospitalization for CHF or emergency visit (ICD 10: I50) (N = 12491)	External	Prevalent and new users Dispensed prescriptions	Current, use in last 14 days before index date (admission date and corresponding date for matched controls)	A: NA B: Rosiglitazone or pioglitazone monotherapy or combination (n = 1692) vs. other OHA monotherapy or combination (n = 87253)
Rajagopalan [51]	Pharmetrics integrated outcomes database USA 1999-2002	Cohort N = 3336 ≥ 18 years	Subjects with one or more claims with ICD-9 code 250.x0 or 250.x2 and/or “evidence of use of antidiabetic medications who began receiving pioglitazone or insulin” during the study period.	First ever, ≥ 1 provider or facility claim with diagnosis of CHF or ≥ 1 inpatient claim with CHF diagnosis (n = NR)	None	New users Dispensed prescriptions	Continuous use for ≥ 90 days of the index therapy	A: NA B: Pioglitazone (n = 1668) vs. insulin as monotherapy or with metformin or SU (n = 1668)
Toprani [52]	USA Veterans Administration 1999-2004	Cohort N = 3956 (only males)	Subjects with one more records with ICD-9 code 250.xx and one or more prescriptions for thiazolidinediones.	First ever, at least one inpatient or outpatient visit with a recorded diagnosis of CHF (ICD-9: 428) (N = 1157)	None	Prevalent and new users Dispensed prescriptions	Users of at least 2 OHAs	A: NA B: TZD vs. non-TZD OHAs (n = not provided)

AHF = acute heart failure; AMI = acute myocardial infarction; CHF = congestive heart failure; DRG = diagnosis-related group; ED = emergency department; GPRD = General Practice Research Database (now the Clinical Practice Research Datalink [CPRD]); HF = heart failure; ICD = *International Classification of Diseases*; ICD-10 = *International Statistical Classification of Diseases and Related Health Problems, 10th Revision*; ICD-9 = *International Classification of Diseases, 9th Revision*; ICD-9-CM = *International Classification of Diseases, 9th Revision, Clinical Modification*; NA = not applicable; NHI = National Health Insurance; NR = not reported; OHA = oral hypoglycemic agent; PPV = positive predictive value; SD = standard deviation; SU = sulfonylurea(s); T1DM = type 1 diabetes mellitus; T2DM = type 2 diabetes mellitus; TZD = thiazolidinedione(s); USA = United States of America.

Note: When it is not indicated that the endpoint is the first ever identified, the study included patients with and without prior history of the study endpoint.

All 12 studies contributing data to the meta-analysis were cohort studies, with one reporting the results of a nested case–control analysis. The vast majority of these studies defined the outcome based on hospitalizations. Only 3 studies also included outpatient diagnoses of heart failure in their endpoint definition [33, 39, 42]. All of the studies included the specific code for heart failure (i.e., ICD-9 428) in their definition except one study that focused on acute heart failure [43]. Five studies were restricted to the evaluation of incident heart failure [33, 38, 39, 41, 42]. The number of heart failure events in each study ranged from 320 to 6900. The age ranges of the study populations with type 2 diabetes varied but included only adults; the vast majority of studies included a broad age range, while a few were restricted to age 65 years or older [34, 37, 44]. The definition of exposure varied across studies in terms of length of exposure, drug combinations, drug comparisons, and statistical handling of treatment changes.

### Meta-analysis results with quality assessment

**Figure 2:**
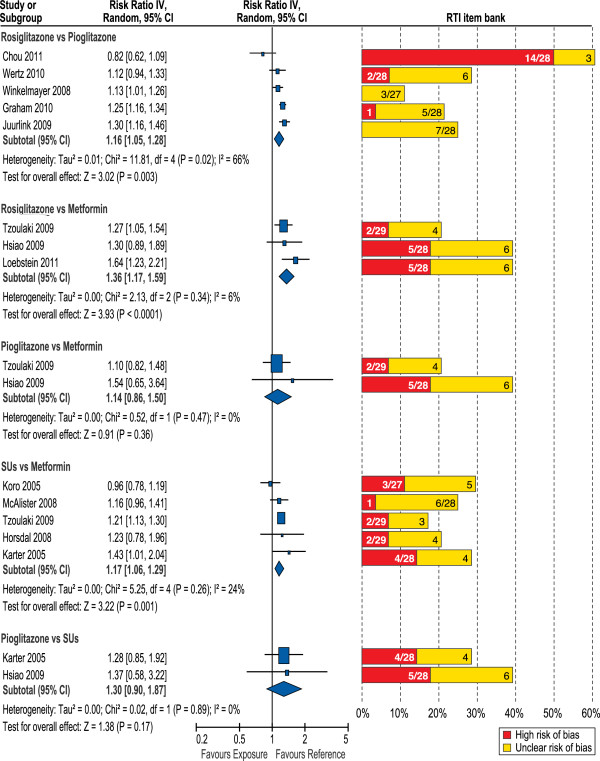
**Heart Failure Relative Risk (Random Effects) in Blood Glucose-Lowering Medications Users and RTI Item Bank Quality Assessment.** IV = inverse variance. Note: For rosiglitazone versus metformin, the study by Tzoulaki et al. [42] contributed with the relative risk reported for combination therapy and for Loebstein et al. [40], we included the fixed-effect summary relative risk for monotherapy and combination therapy (see study reported estimates by subgroup in the Additional file 1). For sulfonylureas versus metformin, the study by Tzoulaki et al. [42] contributed with the reported estimate for the second-generation sulfonylureas.

In Figure 2, the left panel displays the random-effect forest plot for all the drug comparisons included in our meta-analysis. Fixed-effect models produced summary estimates of similar magnitude to those of random-effect models. Because we found across-study heterogeneity within some comparisons, we present only the random-effect estimates in the text unless mentioned otherwise. Summary results of study quality assessment with the RTI item bank for these 12 studies are provided in the right panel. Detailed results of the NOS and the RTI item bank for the 20 studies included in the systematic review are available in Additional file 1: Tables S1e through S3e. Overall, scores with the NOS for the 20 studies were between 5 and 9; the median and mode score was 8. With the RTI item bank, 3 studies had a high risk of bias for 25% or more of the items assessed [33, 51, 52]; and 2 additional studies had a high risk of bias for 20% or more of the items assessed [45, 47]. In our assessment, 4 studies had the potential for immortal time bias [33, 36, 38, 45, 52], 8 studies might have been affected by confounding by indication [33, 39, 40, 42, 47, 50–52], and 8 studies had unmeasured confounding [33, 38, 39, 42, 43, 47, 51, 52]. An overview of the experience with and a comparison of the two tools is reported elsewhere [53].

### Risk of heart failure in rosiglitazone users compared with pioglitazone users

Five cohort studies contributed to evaluation of the risk of heart failure in rosiglitazone users compared with pioglitazone users [33, 34, 37, 43, 44] (Figure 2). Patients were included regardless of whether they were using other blood glucose-lowering drugs. The summary relative risk was 1.16 (95% CI, 1.05-1.28). Heterogeneity was present (*I*^*2*^ *=* 66%). Of the five studies, one had high risk of bias for 50% of the items evaluated [33]. It was the only study with a point effect estimate below unity, and it included prevalent users. When excluding this study in the subanalysis restricted to new users [34, 37, 43, 44] (Table 2 and Additional file 1: Figure S1e), *I*^*2*^ went from 66% to 31%. The fixed effects summary relative risk was 1.21 (95% CI, 1.14-1.30) for new users. These studies defined the outcome as hospitalization for heart failure and included patients with or without prior heart failure. Only one study provided results stratified by prior history of the condition [44]. In that study, stratified results suggested that, among patients without a prior history of heart failure, new users of rosiglitazone had a greater risk of heart failure hospitalization than new users of pioglitazone (relative risk, 1.21; 95% CI, 1.03-1.42), and patients with prior heart failure did not experience increased risk (relative risk, 1.04; 95% CI, 0.89-1.22).

**Table 2:** **Risk of heart failure in Rosiglitazone users compared with Pioglitazone users, overall and subgroup analyses**

	Relative risk (95% Confidence interval)		
		Subgroup analyses	
Study (Author, Year)	All users	New users	New users, Aged ≥ 65 years
*Individual Studies*			
Chou [33]	0.82 (0.62-1.09)	—	—
Graham [34]	1.25 (1.16-1.34)	1.25 (1.16-1.34)	1.25 (1.16-1.34)
Juurlink [37]	1.30 (1.16-1.46)	1.30 (1.16-1.46)	1.30 (1.16-1.46)
Wertz [43]	1.12 (0.94-1.33)	1.12 (0.94-1.33)	—
Winkelmayer [44]	1.13 (1.01-1.26)	1.13 (1.01-1.26)	1.13 (1.01-1.26)
*Meta-Analysis*			
Fixed effects, RR	1.20 (1.15-1.27)	1.22 (1.16-1.28)	1.23 (1.17-1.30)
Random effects, RR	1.16 (1.05-1.28)	1.21 (1.14-1.30)	1.23 (1.14-1.32)
Heterogeneity statistics	τ^*2*^ = 0.01	τ^*2*^ = 0.00	τ^*2*^ = 0.00
	*χ* ^*2*^ = 11.81 (df = 4)	*χ* ^*2*^ = 4.35 (df = 3)	*χ* ^*2*^ = 3.33 (df = 2)
	*I* ^*2*^ = 66%	*I* ^*2*^ = 31%	*I* ^*2*^ = 40%

Df = degrees of freedom; RR = relative risk.

The study populations in 2 studies included subjects younger than 65 years or older. The random effects summary relative risk of the 3 studies conducted in patients aged 65 years or older was 1.23 (95% CI, 1.14-1.32) [34, 37, 44] (Table 2).

Overall, based on available studies, risk of heart failure was about 20% greater for users of rosiglitazone than for users of pioglitazone.

### Risk of heart failure in glitazone users compared with metformin users

Based on 3 studies, the summary relative risk for rosiglitazone, as monotherapy or in combination with other blood glucose-lowering agents, versus metformin was 1.36 (95% CI, 1.17-1.59) [36, 40, 42] (Figure 2). The relative risk for monotherapy was 1.42; (95% CI, 0.95-2.13) and for combination therapy was 1.28 (95% CI, 1.08-1.52) (see Additional file 1: Figure S2e). The effect estimates for monotherapy were heterogeneous across the three studies, *I*^*2*^ = 69%. Quality assessment results displayed on the right panel of Figure 2 show that 2 of the 3 studies had high risk of bias for about 20% of items and an unclear risk of bias for an additional 20% of items [36, 40]. Two of these studies evaluated the risk of heart failure in pioglitazone users compared with metformin users, yielding a summary relative risk of 1.14 (95% CI, 0.86-1.50); we included these studies in the overall forest plot for completeness [36, 42]. Overall, based on few studies, risk of heart failure in users of rosiglitazone was about 40% higher than in users of metformin, while the risk in pioglitazone users was closer to the risk observed in metformin users.

### Risk of heart failure in glitazones users compared with sulfonylurea users

Information on the risk of heart failure in glitazone users compared with sulfonylureas users was very scarce. The only study evaluating the risk of heart failure in rosiglitazone users compared with sulfonylureas users reported a relative risk of 1.22 (95% CI, 0.86-1.74) [36]. This estimate was very similar to the summary relative risk of 1.30 (95% CI, 0.90-1.87) observed for pioglitazone users compared with sulfonylurea users [36, 38] (Figure 2). The study by Karter et al. [38], excluded patients with heart failure events within the 3 prior years.

### Risk of heart failure in sulfonylurea users compared with metformin users

Five studies reported on the risk of heart failure in sulfonylureas users compared with metformin users (Figure 2) [35, 38, 39, 41, 42], but only 3 included the list of individual sulfonylureas evaluated [39, 41, 42]. All were cohort studies, and 1 included a nested case–control analysis. One study presented the effect estimates for treatment periods of first-generation and second-generation sulfonylureas separately [42]. For our main analysis, we included the reported relative risk for second-generation sulfonylureas as they were most frequently used. The overall random effect summary relative risk was 1.17 (95% CI, 1.06-1.29). Heterogeneity of effect estimates across studies did not look important (*I*^*2*^ = 24%). There was high risk of bias for about 15% or less of the assessed items, but in some studies, the risk of bias was unclear for an additional 10% to 20% of the items. When in the analysis we included the reported relative risk for the first-generation sulfonylureas in the Tzoulaki et al. study instead of that reported for the second-generation, the overall random effect summary relative risk was 1.20 (95% CI, 1.04-1.39) [42], but with substantial heterogeneity (*I*^*2*^ = 53%).

The definition of current exposure varied largely across studies, from use at index date [38, 42] to use during the last 90 days prior to the index date or presence of at least 1 prescription in the study period [41]. Random effects summary estimates remained similar to those of the overall analysis, when restricting analysis to studies including only incident events (relative risk, 1.16; 95% CI, 1.03-1.31; n = 4) or reporting only on monotherapy regimens (relative risk, 1.15; 95% CI, 1.04-1.28; n = 4) (Table 3). Only 2 studies were restricted to new users, yielding a summary relative risk of 1.22 (95% CI, 1.02-1.46) (Table 3 and Additional file 1: Figure S3e) [38, 41].

**Table 3:** **Risk of heart failure in sulfonylurea users compared with metformin users, overall and subgroup analyses**

	Relative risk (95% confidence interval)					
			Subgroup analyses			
Study Author, Year	Overall	Cohort studies	Monotherapy ^a^	Incident heart failure	New users	New or prevalent users
**Individual Studies**						
Koro [39]	0.96 (0.78, 1.19)	—	0.96 (0.78, 1.19)	0.96 (0.78, 1.19)	—	0.96 (0.78, 1.19)
McAlister [41]	1.16 (0.96, 1.41)	1.16 (0.96, 1.41)	1.16 (0.96, 1.41)	1.16 (0.96, 1.41)	1.16 (0.96, 1.41)	—
Horsdal [35]	1.23 (0.78, 1.96)	1.23 (0.78, 1.96)	1.23 (0.78, 1.96)	—	—	1.23 (0.78, 1.96)
Tzoulaki [42]	1.21 (1.13, 1.30)	1.21 (1.13, 1.30)	1.21 (1.13, 1.30)	1.21 (1.13, 1.30)	—	1.21 (1.13, 1.30)
Karter [38]	1.43 (1.01, 2.04)	1.43 (1.01, 2.04)	—	1.43 (1.01, 2.04)	1.43 (1.01, 2.04)	—
*Meta-Analysis*						
Fixed-effects, RR	1.19 (1.12, 1.26)	1.21 (1.14, 1.29)	1.18 (1.11, 1.26)	1.19 (1.12, 1.26)	1.22 (1.03, 1.44)	1.18 (1.11, 1.27)
Random effects, RR	1.17 (1.06, 1.29)	1.21 (1.14, 1.29)	1.15 (1.04, 1.28)	1.16 (1.03, 1.31)	1.22 (1.02, 1.46)	1.13 (0.95, 1.33)
Heterogeneity statistics	τ^2^ = 0.00	τ^2^ = 0.00	τ^2^ = 0.00	τ^2^ = 0.01	τ^2^ = 0.00	τ^2^ = 0.01
	*χ* ^2^ = 5.25	*χ* ^2^ = 1.06	*χ* ^2^ = 4.15	*χ* ^2^ = 5.22	*χ* ^2^ = 1.04	*χ* ^2^ = 4.11
	(df = 4)	(df = 3)	(df = 3)	(df = 3)	(df = 1)	(df = 2)
	I^2^ = 24%	I^2^ = 0%	I^2^ = 28%	I^2^ = 43%	I^2^ = 4%	I^2^ = 51%

Df = degrees of freedom; RR = relative risk.

^a^Sulfonylurea and metformin in monotherapy.

One study reported the effect of dose among users of sulfonylureas and metformin. Monotherapy sulfonylurea users exposed to high doses (defined as higher than the median daily dose for each individual medication) were more likely to develop heart failure than those using lower doses (hazard ratio, 1.38; 95% CI, 1.20-1.60) [41]. For metformin, the hazard ratio was 1.06 (95% CI, 0.81-1.41) comparing doses higher than the median daily dose versus lower doses.

Overall, risk of heart failure in sulfonylurea users was about 20% higher than the risk in metformin users.

### Publication bias

Examination of the funnel plots does not suggest publication bias, although the number of studies was small for all the comparisons evaluated. The funnel plot for studies (n = 5) evaluating the risk of heart failure in rosiglitazone users compared with pioglitazone users and in sulfonylurea users compared with metformin users are both displayed in Additional file 1. The apex in each funnel plot is pointing up around a relative risk of 1.3 for each of these two comparisons (Additional file 1: Figures S4e and S5e).

## Discussion

This systematic review of published observational studies on the risk of heart failure associated with noninsulin blood glucose-lowering drugs in patients with T2DM confirmed that studies in this field are very heterogeneous. Therefore, summarizing the scientific evidence is challenging. The lack of a common reference medication for evaluation of all potential exposures across studies limited direct comparison of effect estimates. Of the 20 studies included in our systematic literature review, only 12 could contribute to the meta-analysis. Our summary effect estimates are compatible with a small increase in the risk of heart failure among patients with T2DM using rosiglitazone compared with the risk in those using pioglitazone. Users of glitazones were included whether or not they were reported to use other blood glucose-lowering drugs. Both glitazone users and sulfonylurea users seem to be at increased risk of heart failure compared with metformin users. Observational studies evaluating the risk of heart failure of newer blood glucose-lowering agents were lacking. Such studies are anticipated in light of the recent unexpected results of the SAVOR_TIMI 53 trial reporting that more patients in the saxagliptin group were hospitalized for heart failure than in the placebo group (relative risk: 1.27; 95% CI, 1.07-1.51) [54]. A recent publication reported on sitagliptin users from an observational study performed in a large USA claims database among patients with diabetes and heart failure. Patients using sitagliptin presented higher risk of hospital readmission for heart failure than non–sitagliptin users [55]. However, this study was not eligible for our meta-analysis because the reference group includes all other glucose-lowering agents combined.

The results of this meta-analysis are consistent with published clinical trial information. A prior meta-analysis of randomized, controlled clinical trials reported that patients randomized to glitazones had a 2-fold greater risk of heart failure than patients randomized to placebo [56]. A large randomized clinical trial in patients with T2DM and preexisting cardiovascular disease, the PROactive study, reported that patients on pioglitazone had serious heart failure events more frequently than those on placebo [57]. In the RECORD clinical trial, patients on monotherapy with metformin or sulfonylureas who were randomized to add-on rosiglitazone had twice the risk of heart failure than those randomized to a combination of metformin with sulfonylurea [58].

Most of the published meta-analyses of observational studies have focused on patients with diabetes and heart failure and concluded that glitazones are contraindicated in these patients, while metformin can be safely used [21, 59]. A meta-analysis of observational studies performed in patients with T2DM and treated with glitazones reported a risk of heart failure in users of rosiglitazone almost 20% higher than the risk in users of pioglitazone [24].

### Heterogeneity and subgroup analyses

Although the studies included in our meta-analysis were heterogeneous, all of the subgroup analyses performed were compatible with the overall results. For example, in the comparison of rosiglitazone and pioglitazone, the analysis restricted to new users resulted in an effect estimate of 1.21 (95% CI, 1.14-1.30), very close to the overall hazard ratio of 1.16 (Table 2). The inclusion of prevalent users can introduce bias caused by the underascertainment of events that occur after the start of the therapy but before the study start, which may lead to depletion of susceptible patients, and by the inability to control for risk factors that may be modified by the study drugs [60, 61].

The conducted subgroup analyses consistently suggest a greater occurrence of heart failure in rosiglitazone users than in pioglitazone users.

### Heart failure ascertainment

The majority of included studies defined the outcome based on hospitalizations, and all except one study included the ICD-9 428 discharge code. The positive predictive value of ICD-9 code 428 as a primary diagnosis has been reported to be 94.3% using the Framingham criteria and 88.6% using criteria previously validated with pulmonary capillary wedge pressure [62]. Since heart failure is a complex and progressive syndrome whose recognition can be obscured by a number of differential diagnoses, in studies without internal case validation we advise the use of specific primary discharge codes for heart failure hospitalizations. Because of the progressive evolution from the initial symptoms and signs to overt congestive heart failure, the onset date of heart failure is difficult to determine in the absence of outpatient diagnoses. If relying only on hospitalizations, severe events or heart failure exacerbations will be captured, but milder episodes will be missed because those episodes are diagnosed and stabilized in the outpatient setting, most frequently in the elderly. Since less than half of the studies were restricted to the evaluation of incident heart failure [33, 38, 39, 41, 42], the other studies might be affected by selection biases such as contraindication bias due to chronic heart failure. For example, metformin used to be contraindicated in patients with heart failure, and risk effect estimates relative to metformin use in studies from that time might be potentially biased toward the null or be negatively confounded. Our summary effect estimate comparing metformin and sulfonylurea users was not affected by this type of bias, since 4 of the 5 included studies evaluated incident events, and the analysis restricted to these 4 studies yielded the same effect estimate (Table 3).

### Role of biases in included studies

Observational studies are vulnerable to confounding and selection and information biases; therefore, limitations of the studies included in this meta-analysis could have affected our main results.

#### Depletion of susceptibles

The reported low effect estimate of 0.82 (95% CI, 0.62-1.09) for rosiglitazone users compared with pioglitazone users in one study [33] may be related to several biases, including depletion of susceptibles. In this cohort study, patients were included only if they were treated with glitazones (prevalent or incident use) for more than 120 days within the first 180 days after the date of the prescription. If rosiglitazone is associated with a greater cardiovascular risk than pioglitazone, as is suggested by our findings, and prevalent users are included, differential depletion of susceptible patients between the rosiglitazone and pioglitazone groups might account for the apparent beneficial effect seen in rosiglitazone users compared with pioglitazone users. That is, more rosiglitazone users than pioglitazone users might have been excluded from the study within the 120-day use criterion due to early cardiovascular side effects.

#### Clinical guidelines and prescription patterns

The application of clinical guidelines could have operated in opposite directions over the years, as recommendations changed over time. Common to all issued clinical guidelines is the fact that the treatment of diabetes is guided by disease severity and patient characteristics. Few studies have accounted for disease severity; therefore, confounding by indication might be present in the majority of the within-study comparisons of this meta-analysis. Most of the comparisons with metformin could be affected by this type of bias because metformin is recommended as first-line therapy and is indicated for patients with less severe disease. This might be relevant for the comparisons between sulfonylurea and metformin users. The cardiovascular concerns for sulfonylureas dated back to the 1970s when the University Group Diabetes Program (UGDP) was terminated prematurely because of an excess of cardiovascular mortality associated with tolbutamide (the first-generation sulfonylurea used in the study). A special warning on the potentially increased risk of cardiovascular mortality associated with all sulfonylureas was issued in the USA. The results of the UK Prospective Diabetes Study (UKPDS), comparing the study sulfonylureas (chlorpropamide, glibenclamide, or glipizide) with insulin, showed that the intensive blood-glucose control with either sulfonylureas or insulin decreases the risk of microvascular complications, but not macrovascular disease, in patients with type 2 diabetes. None of the individual drugs had an adverse effect on cardiovascular outcomes. Later published observational studies on the associated risk of cardiovascular outcomes with the use of sulfonylureas have been heterogeneous and with variability on the individual sulfonylureas evaluated. Therefore, the cardiovascular safety of sulfonylureas remains unclear.

As mentioned previously, the 2012 joint clinical guidelines of the ADA and the EASD emphasized that the choice of blood glucose-lowering agent should focus on drug safety, especially protecting against hypoglycemia, heart failure, renal dysfunction, bone fractures, and drug-drug interactions [15]. However, clinical guidelines have been changing over time. Metformin and rosiglitazone were previously contraindicated in patients with heart failure and were avoided to some extent in patients at high risk of developing heart failure. At present, metformin is being recommended as first-line therapy in clinically stable patients with heart failure if their ventricular dysfunction is not severe. Metformin is not recommended in patients with severe renal or hepatic impairment because of the risk of lactic acidosis. The cardiovascular safety of newer blood glucose-lowering medications is less known [63]. As a consequence of the aging of the population overall and improved survival after myocardial infarction, patients with T2DM and progressive heart failure are now frequently seen in clinical practice. The medical care of these patients is challenging; their multiple comorbidities often require treatment with several concomitant medications and determine contraindications to various blood glucose–lowering agents. Avoiding glitazones among patients at high risk of or with existing heart failure is recommended in clinical guidelines for both diabetes treatment and heart failure control. Glitazones are known to cause sodium and water retention and increased risk of worsening heart failure and hospitalization. Because of the impact of clinical guidelines on prescribing patterns, the risk of heart failure associated with metformin and glitazones might be underestimated in the evaluated published studies.

#### Formulary restrictions

A similar bias is that imposed by formulary restrictions in the health plans from which many of the study populations were drawn. For example, because at Maccabi Healthcare Services rosiglitazone could be prescribed only if there was an inadequate control of blood glucose with sulfonylurea, metformin, or both, the heart failure effect estimate for rosiglitazone versus metformin monotherapy in the study by Loebstein et al. [40] (relative risk, 2.23; 95% CI, 1.41-3.53) might be overestimated.

#### Residual confounding

Residual confounding can be a major limitation for the majority of the included studies since the magnitude of the increased risks was rather small for most of the comparisons. Residual confounding might be present in studies that failed to systematically record some lifestyle factors. Few studies adjusted for socioeconomic status or education and physical activity, which can all be associated with both treatment selection and the development of outcome. Metformin has been the medication of choice for obese patients with T2DM since the results of the United Kingdom Prospective Diabetes Study showed a beneficial effect on the risk of myocardial infarctions and diabetes-related deaths of initial therapy with metformin in overweight and obese patients [64]. The majority of the studies included in our meta-analysis failed to completely adjust for indicators of obesity, which is highly prevalent among the T2DM population. Due to selective prescribing of metformin to overweight/obese patients with T2DM and the known increased risk of heart failure among obese patients, a higher absolute risk of heart failure is expected among these patients. This could bias the relative risk estimate towards the null when other blood glucose-lowering agents are compared with metformin.

#### Intermediate factors

Glycemic indicators, such hemoglobin A1_C_, can be considered causal intermediate factors since they are related to the effectiveness of the blood glucose-lowering drugs and to the severity of diabetes, which is a risk factor for cardiovascular complications. Several studies adjusted for blood glucose indicators during patient follow-up, which could underestimate the relative risk [33, 35, 40, 48, 52]. Further, if they are considered potential time-varying confounders, appropriate statistical techniques should have been applied [65]. For example, if levels of hemoglobin A1_C_ are measured periodically and determine treatment decisions and future risk of heart failure, as treatment will also modify the levels of hemoglobin A1_C_, use of statistical techniques to account for this relationship is warranted.

### Dose and duration effects

We were not able to evaluate the effect of dose and duration due to the scarcity of data in the published studies. One study reported that sulfonylurea used at high doses increased the risk of heart failure compared with lower doses while results for metformin were inconclusive [41]. Since blood glucose-lowering agents should be initiated at low doses and titrated up according to the degree of glycemic control, clinical guidelines recommend tailoring treatment to each patient. Therefore, dose and treatment duration of each individual medication and add-on medications are very important to evaluate safety.

### Literature search update

In September 2014, we updated the literature search using the original search terms in PubMed. Embase and the Cochrane Library, which had not identified any studies to include in the original systematic review beyond those found in PubMed, were not searched in this update. Three new studies were considered to be eligible and were reviewed [66–68]; all provided results on the comparison of rosiglitazone and pioglitazone users, and one compared glitazone users with metformin users [68]. The updated effect estimate for rosiglitazone compared with pioglitazone users remained practically unchanged, with a summary relative risk of 1.16 (95% CI, 1.03-1.30). For the comparison with metformin, the updated summary relative risk was 1.42 (95% CI, 1.21-1.66) for rosiglitazone and 1.12 (95% CI, 0.88-1.43) for pioglitazone, very similar to the values obtained in the original meta-analysis. A few more studies were reviewed [55, 69–71], but the reference groups without the medication of interest included all the other medications combined and therefore were not eligible for our meta-analysis.

### Strengths and limitations

Our systematic review was carefully planned, and we included in the meta-analysis only studies with a clear definition of the reported comparisons, which should be useful for clinicians making decisions on specific therapeutic alternatives for specific patients. In addition, our detailed evaluation of the quality of each reviewed study using the NOS and the RTI item bank helps to interpret the results of heterogeneous studies evaluating the risk of heart failure associated with each individual medication from classes that are also very heterogeneous in their mechanisms of action.

The main limitation of this meta-analysis was the heterogeneity in the design of the primary studies. Key drivers of this heterogeneity were the complex array of treatment options based on guidelines, formularies, varying diabetes severity status, and the outcome definitions. The studies that combined medications (e.g., “any other treatment”) as the comparison group are of particular concern for this and future meta-analyses for two main reasons. First, “any other treatment” represents different treatments depending on the study period and population, which decreases the applicability and comparability of results. Second, results relative to such reference treatment may not be useful for clinical decision makers who need to choose between specific therapeutic alternatives. In addition, the lack of specific comparisons to evaluate the risk of heart failure in users of each individual sulfonylurea makes difficult the interpretation of our findings for this heterogenous group of medications. Further studies of use of individual sulfonylureas on the risk of heart failure are warranted.

## Conclusions

In conclusion, the overall results of this meta-analysis suggest that patients with T2DM using either glitazones or sulfonylureas might be at greater risk of heart failure than metformin users. However, indication bias might account for part of the differential effect between these medications.

Results from ongoing large multidatabase studies, carefully planned and conducted, are awaited and will help to elucidate the risk of heart failure in patients with diabetes. These studies will evaluate newer available blood glucose-lowering therapies.

## Electronic supplementary material

Additional file 1: **Online Appendix.** **Table S1e.** Search Terms for Medline Search. **Table S2e.** Newcastle-Ottawa Scale Quality Assessment Results, Individual Case–control Studies. **Table S3e.** Newcastle-Ottawa Scale Quality Assessment Results, Individual Cohort Studies. **Table S4e.** RTI Item Bank Adapted to the Present Systematic Review. **Table S5e.** RTI Item Bank Quality Assessment Results, Individual Studies. **Figure S1e.** Heart Failure Relative Risk (Random Effects) in New Users: Rosiglitazone Compared With Pioglitazone. **Figure S2e.** Heart Failure Relative Risk (Random Effects) in Rosiglitazone Users Compared With Metformin Users, by Type of Regimen. **Figure S3e.** Heart Failure Relative Risk (Random Effects) in Sulfonylurea Users Compared With Metformin Users, by Type of Use. **Figure S4e.** Funnel Plot: Heart Failure Relative Risk for Rosiglitazone Users Compared With Pioglitazone Users (5 Studies). **Figure S5e.** Funnel Plot: Heart Failure Relative Risk for Sulfonylureas Users Compared With Metformin Users (5 Studies). MOOSE Checklist. (DOC 802 KB)

## Abbreviations

hemoglobin A1_C_: glycated hemoglobin
ADA: American Diabetes Association
CI: confidence interval
EASD: European Association for the Study of Diabetes
EMA: European Medicines Agency
FDA: Food and Drug Administration
I^2^: heterogeneity statistic interpreted as the percentage of variability on a set of effect size estimates due to heterogeneity between studies rather than sampling error
ICD-9: International Classification of Diseases 9th Revision
MeSH: medical subject heading
NOS: Newcastle-Ottawa Quality Assessment Scale
SAFEGUARD: Safety Evaluation of Adverse Reactions in Diabetes (project)
T2DM: type 2 diabetes mellitus
USA: United States of America.
