# Supplementary material for: The risk of heart failure associated with the use of noninsulin blood glucose-lowering drugs: systematic review and meta-analysis of published observational studies
Source: BMC Cardiovasc Disord. 2014 Sep 26;14:129. doi: 10.1186/1471-2261-14-129 (PMC4246471; doi:10.1186/1471-2261-14-129)
Supplement: Supplementary file 1 — Additional file 1: Online Appendix. Table S1e. Search Terms for Medline Search. Table S2e. Newcastle-Ottawa Scale Quality Assessment Results, Individual Case–control Studies. Table S3e. Newcastle-Ottawa Scale Quality Assessment Results, Individual Cohort Studies. Table S4e. RTI Item Bank Adapted to the Present Systematic Review. Table S5e. RTI Item Bank Quality Assessment Results, Individual Studies. Figure S1e. Heart Failure Relative Risk (Random Effects) in New Users: Rosiglitazone Compared With Pioglitazone. Figure S2e. Heart Failure Relative Risk (Random Effects) in Rosiglitazone Users Compared With Metformin Users, by Type of Regimen. Figure S3e. Heart Failure Relative Risk (Random Effects) in Sulfonylurea Users Compared With Metformin Users, by Type of Use. Figure S4e. Funnel Plot: Heart Failure Relative Risk for Rosiglitazone Users Compared With Pioglitazone Users (5 Studies). Figure S5e. Funnel Plot: Heart Failure Relative Risk for Sulfonylureas Users Compared With Metformin Users (5 Studies). MOOSE Checklist. (DOC 802 KB) [file 12872_2014_805_MOESM1_ESM.doc]

Additional file 1: Online Appendix

Contents

[Table S1e. Search terms for Medline search 2](#__RefHeading___Toc391023151)

[Table S2e. Newcastle-Ottawa Scale Quality Assessment Results, Individual Case-Control Studies 5](#__RefHeading___Toc391023152)

[Table S3e. Newcastle-Ottawa Scale Quality Assessment Results, Individual Cohort Studies 6](#__RefHeading___Toc391023153)

[Table S4e. RTI Item Bank Adapted to the Present Systematic Review 7](#__RefHeading___Toc391023154)

[Table S5e. RTI Item Bank Quality Assessment Results, Individual Studies 9](#__RefHeading___Toc391023155)

[Figure S1e. Relative Risk of Heart Failure in New Users of Rosiglitazone Compared With the Risk in New Users of Pioglitazone, Results From Published Studies and Summary Relative Risk by Random Effects 10](#__RefHeading___Toc391023156)

[Figure S2e. Relative Risk of Heart Failure in Rosiglitazone Users Compared With the Risk in Metformin Users; Results From Published Studies by Type of Regimen and Summary Relative Risks by Random Effects 11](#__RefHeading___Toc391023157)

[Figure S3e. Relative Risk of Heart Failure in Sulfonylurea Users Compared With the Risk in Metformin Users; Results From Published Studies and Summary Relative Risk by Random Effects 12](#__RefHeading___Toc391023158)

[Figure S4e. Funnel Plot of the Relative Risk of Heart Failure for Rosiglitazone Users Compared With Pioglitazone Users (5 Studies) 13](#__RefHeading___Toc391023159)

[Figure S5e. Funnel Plot of the Relative Risk of Heart Failure for Sulfonylureas Users Compared With Metformin Users (5 Studies) 14](#__RefHeading___Toc391023160)

[MOOSE Checklist 15](#__RefHeading___Toc391023161)

Table Se. Search terms for Medline search

| Search Number | Description | Input Terms |
| --- | --- | --- |
| #1 | Drug name search | "glyburide"[Mesh] OR "glyburide"[All Fields] OR "glibenclamide"[All Fields] OR "gliclazide"[Mesh] OR "gliclazide"[All Fields] OR "glipizide"[Mesh] OR "glipizide"[All Fields] OR "glimepiride"[Supplementary Concept] OR "glimepiride"[All Fields] OR "tolbutamide"[Mesh] OR "tolbutamide"[All Fields] OR "metformin"[Mesh] OR "metformin"[All Fields] OR "rosiglitazone"[Supplementary Concept] OR "rosiglitazone"[All Fields] OR "pioglitazone"[Supplementary Concept] OR "pioglitazone"[All Fields] OR "acarbose"[Mesh] OR "acarbose"[All Fields] OR "exenatide"[Supplementary Concept] OR "exenatide"[All Fields] OR "liraglutide"[Supplementary Concept] OR "liraglutide"[All Fields] OR "sitagliptin"[Supplementary Concept] OR "sitagliptin"[All Fields] OR "vildagliptin"[Supplementary Concept] OR "vildagliptin"[All Fields] OR "saxagliptin"[Supplementary Concept] OR "saxagliptin"[All Fields] OR "BI 1356"[All Fields] OR "Linaclotide Acetate"[All Fields] OR "linagliptin"[All Fields] OR "repaglinide"[Supplementary Concept] OR "repaglinide"[All Fields] OR "nateglinide"[Supplementary Concept] OR "nateglinide"[All Fields] OR "acetohexamide"[Mesh] OR "acetohexamide"[All Fields] OR "chlorpropamide"[Mesh] OR "chlorpropamide"[All Fields] OR "carbutamide"[Mesh] OR "carbutamide"[All Fields] OR "tolazamide"[Mesh] OR "tolazamide"[All Fields] OR "glyclopyramide"[All Fields] OR "voglibose"[Supplementary Concept] OR "voglibose"[All Fields] OR "miglitol"[Supplementary Concept] OR "miglitol"[All Fields] OR "pramlintide"[Supplementary Concept] OR "pramlintide"[All Fields] OR "alogliptin"[Supplementary Concept] OR "alogliptin"[All Fields] OR "benfluorex"[Supplementary Concept] OR "benfluorex"[All Fields] OR "miglitinide"[All Fields] OR "meglitinide"[Supplementary Concept] OR "meglitinide"[All Fields] |
| #2 | Chemical or pharmacolo­gical class names | "sulfonylurea compounds"[Mesh] OR "sulfonylurea"[All Fields] OR "sulphonylurea"[All Fields] OR "biguanides"[Mesh] OR "biguanide"[All Fields] OR "thiazolidinediones"[Mesh] OR thiazolidinedione*[All Fields] OR "glitazone"[All Fields] OR "glitazones"[All Fields] OR (("alpha-glucosidases"[Mesh] OR "alpha-glucosidase"[All Fields] OR ("alpha"[All Fields] AND "glucosidase"[All Fields]) OR "alpha glucosidase"[All Fields]) AND ("antagonists and inhibitors"[Subheading] OR "antagonist"[All Fields OR "antagonists"[All Fields] OR “inhibitor"[All Fields] OR “inhibitors"[All Fields])) OR (("glucagon-like peptide receptor"[Supplementary Concept] OR "glucagon-like peptide receptor"[All Fields] OR "glucagon like peptide 1 receptor"[All Fields]) AND ("agonists"[Subheading] OR "agonist"[All Fields] OR "agonists"[All Fields])) OR "dipeptidyl-peptidase iv inhibitors"[Mesh] OR ("dipeptidyl-peptidase"[All Fields] AND "iv"[All Fields] AND "inhibitor"[All Fields]) OR "dipeptidyl-peptidase iv inhibitors"[All Fields] OR "dipeptidyl peptidase 4 inhibitors"[All Fields] OR "dipeptidyl-peptidase iv inhibitors"[Pharmacological Action] OR ((("PPAR gamma"[Mesh] OR ("ppar"[All Fields] AND "gamma"[All Fields]) OR "ppar gamma"[All Fields] OR ("peroxisome"[All Fields] AND "proliferator"[All Fields] AND "activated"[All Fields] AND "receptor"[All Fields] AND "gamma"[All Fields]) OR "peroxisome proliferator activated receptor gamma"[All Fields])) AND ("agonists"[Subheading] OR "agonist"[All Fields] OR "agonists"[All Fields])) OR "incretins"[Mesh] OR "incretins"[All Fields] OR "incretins"[Pharmacological Action] |
| #3 | Broader terms | "hypoglycemic agents"[Mesh] OR "hypoglycemic agent"[All Fields] OR "hypoglycemic agents"[All Fields] OR "hypoglycemic agents"[Pharmacological Action] OR "hypoglycaemic agent"[All Fields] OR "hypoglycaemic agents"[All Fields] OR "hypoglycemic drug"[All Fields] OR "hypoglycemic drugs"[All Fields] OR "hypoglycaemic drug"[All Fields] OR "hypoglycaemic drugs"[All Fields] OR "antidiabetic"[All Fields] |
| #4 | OR statement | #1 OR #2 OR #3 |
| #5 | Clinical outcome terms | "Myocardial Infarction"[Mesh] OR myocardial infarction*[Text Word] OR heart attack*[Text Word] OR "Heart Failure"[Mesh] OR heart failure*[Text Word] OR "Arrhythmias, Cardiac"[Mesh] OR arrhythmia*[Text Word] OR "Death, Sudden, Cardiac"[Mesh] OR sudden cardiac death*[Text Word] OR "Brain Ischemia"[Mesh] OR "Intracranial Hemorrhages"[Mesh] OR "Stroke"[Mesh] OR stroke*[Text Word] OR "Cerebrovascular Disorders"[Mesh] OR "cerebrovascular"[Title] OR "cardiovascular"[Title] |
| #6 | Cardiovas­cular mortality outcome terms | "Myocardial Infarction/mortality"[Mesh] OR "Heart Failure/mortality"[Mesh] OR "Arrhythmias, Cardiac/mortality"[Mesh] OR "Death, Sudden, Cardiac/epidemiology"[Mesh] OR "Brain Ischemia/mortality"[Mesh] OR "Intracranial Hemorrhages/mortality"[Mesh] OR "Stroke/mortality"[Mesh] OR "Cerebrovascular Disorders/mortality"[Mesh] OR "Cardiovascular Diseases/mortality"[Mesh] OR sudden cardiac death*[Text Word] OR "cerebrovascular mortality"[Text Word] OR cerebrovascular death*[Text Word] OR "cardiovascular mortality"[Text Word] OR cardiovascular death*[Text Word] |
| #7 | OR statement | #5 OR #6 |
| #8 | Filter terms for observational studies | "Epidemiologic Studies"[Mesh] OR "epidemiologic study"[All Fields] OR "epidemiological study"[All Fields] OR "Cohort Studies"[Mesh] OR ("cohort"[All Fields] AND "study"[All Fields]) OR "cohort study"[All Fields] OR "Risk"[Mesh] OR "Case-Control Studies"[Mesh] OR ("case-control"[All Fields] AND "study"[All Fields]) OR "case-control study"[All Fields] OR ("case"[All Fields] AND "control"[All Fields] AND "study"[All Fields]) OR "case control study"[All Fields] OR "Longitudinal Studies"[Mesh] OR "longitudinal study"[All Fields] OR ("longitudinal"[All Fields] AND "study"[All Fields]) OR "Retrospective Studies"[Mesh] OR ("retrospective"[All Fields] AND "study"[All Fields]) OR "retrospective study"[All Fields] OR "observational"[All Fields] OR "cohort"[All Fields] OR ("case"[All Fields] AND "control"[All Fields]) OR "Cross-Sectional Studies"[Mesh] OR "cross sectional"[All Fields] OR "non-randomized"[All Fields] OR "nonrandomized"[All Fields] OR "non-randomised"[All Fields] OR "nonrandomised"[All Fields] |
| #9 | AND  statement | #4 AND #7 AND #8 |
| #10 | Filter to exclude non- systematic reviews | "Review"[Publication Type] NOT ("Meta-Analysis"[Publication Type] OR "Meta- Analysis as Topic"[Mesh] OR "meta-analysis"[All Fields] OR systematic[sb] OR systematic review*[All Fields] OR "Comparative Effectiveness Research"[Mesh] OR comparative effect*[All Fields]) |
| #11 | Filter for other publication types | "Letter"[Publication Type] OR "Comment"[Publication Type] OR "Randomized Controlled Trial"[Publication Type] OR "Editorial"[Publication Type] |
| #12 | NOT  statement | #9 NOT (#10 OR #11) |
| #13 | Statement to exclude animal studies | "Animals"[Mesh] NOT "Humans"[Mesh] |
| #14 | Final | #12 NOT #13 |

Table Se. Newcastle-Ottawa Scale Quality Assessment Results, Individual Case-Control Studies

|  |  | Domain and Topics | | | | | | | | |  |
| --- | --- | --- | --- | --- | --- | --- | --- | --- | --- | --- | --- |
|  |  | Selection | | | | Comparability | | Exposure | | |  |
| First Author | Year | Is the Case Definition Adequate? | Representativeness of the Cases | Selection of Controls | Definition of Controls | Comparability: Age and Sex | Comparability: Additional Factors | Ascertainment of Exposure | Ascertainment for Cases and Controls | Non-response Rate for Cases and Controls | Total |
| Hartung | 2005 | ★ | ★ |  |  | ★ |  | ★ | ★ |  | 5 |
| Koroa | 2005 | ★ | ★ | ★ |  | ★ | ★ | ★ | ★ |  | 7 |
| Lipscombe | 2007 | ★ | ★ | ★ | ★ | ★ | ★ | ★ | ★ |  | 8 |

^a^ Included in meta-analysis.

Table Se. Newcastle-Ottawa Scale Quality Assessment Results, Individual Cohort Studies

|  |  | Domain and Topic | | | | | | | | |  |
| --- | --- | --- | --- | --- | --- | --- | --- | --- | --- | --- | --- |
|  |  | Selection | | | | Comparability | | Outcome | | |  |
| Author | Year | Representativeness of the Exposed Cohort | Selection of the Non-Exposed Cohort | Ascertainment of Exposure | Outcome Was Not Present at Study Start | Comparability: Age and Sex | Comparability: Additional Factors | Assessment of Outcome | Was Follow-Up Long Enough for Outcomes to Occur? | Adequacy of Follow -up of Cohorts | Total |
| Chou^a^ | 2011 |  | ★ | ★ | ★ | ★ | ★ | ★ | ★ | ★ | 8 |
| Delea | 2003 | ★ |  | ★ | ★ | ★ | ★ | ★ | ★ |  | 7 |
| Graham^a^ | 2010 | ★ | ★ | ★ | ★ | ★ | ★ | ★ |  | ★ | 8 |
| Habib | 2009 | ★ | ★ | ★ |  | ★ | ★ | ★ | ★ | ★ | 8 |
| Horsdal^a^ | 2008 | ★ | ★ | ★ | ★ | ★ | ★ | ★ | ★ | ★ | 9 |
| Horsdal | 2009 | ★ | ★ | ★ | ★ | ★ | ★ | ★ | ★ | ★ | 9 |
| Hsiao^a^ | 2009 | ★ | ★ | ★ |  | ★ | ★ | ★ | ★ |  | 7 |
| Hsiao | 2010 | ★ | ★ | ★ |  | ★ | ★ | ★ | ★ |  | 7 |
| Juurlink^a^ | 2009 | ★ | ★ | ★ |  | ★ | ★ | ★ |  | ★ | 7 |
| Karter^a^ | 2005 | ★ | ★ | ★ | ★ | ★ | ★ | ★ |  | ★ | 8 |
| Loebstein^a^ | 2011 | ★ | ★ | ★ |  | ★ | ★ | ★ | ★ | ★ | 8 |
| McAlister^a^ | 2008 |  | ★ | ★ | ★ | ★ | ★ | ★ | ★ |  | 7 |
| Rajagopalan | 2004 | ★ | ★ | ★ | ★ | ★ | ★ | ★ | ★ |  | 8 |
| Toprani | 2011 |  | ★ | ★ | ★ | ★ | ★ |  | ★ |  | 6 |
| Tzoulaki^a^ | 2009 | ★ | ★ | ★ |  | ★ | ★ | ★ | ★ | ★ | 8 |
| Wertz^a^ | 2010 | ★ | ★ | ★ |  | ★ | ★ | ★ | ★ | ★ | 8 |
| Winkelmayer^a^ | 2008 | ★ | ★ | ★ | ★ | ★ | ★ | ★ | ★ | ★ | 9 |

^a^ Included in meta-analysis

Table S4e. RTI Item Bank Adapted to the Present Systematic Review

| Domains and Items (Numbered) |
| --- |
| Study population definition and selection |
| 1a. Prospective/retrospective design: potential for recall bias |
| 1b. Prospective/retrospective design: tailored data collection |
| 2. Critical inclusion/exclusion criteria: clearly stated? |
| 3. Critical inclusion/exclusion criteria: valid and reliable measures? |
| 4. Critical inclusion/exclusion criteria: applied uniformly? |
| 5. Strategy for recruitment: same across study groups |
| 6. Precision |
| Exposure |
| 7. Level of detail in describing the exposure |
| Outcome |
| 8. Important outcomes prespecified? |
| Exposure groups |
| 9. Selection of the comparison group adequate? |
| 10. Allocation between the groups: balance |
| 11. Isolation from unintended exposures |
| Blinding |
| 12. Outcome validation independent of exposure status |
| Soundness of information |
| 13. Exposures: valid and reliable measures, consistently implemented? |
| 14a. 14a. Outcomes: valid and reliable measures? |
| 14b. 14b. Outcomes: measures consistently implemented? |
| Follow-up |
| 15. Length of follow-up: same for all groups? |
| 16. Length of follow-up: long enough? |
| 17. Attrition: different across exposure groups |
| Analysis comparability |
| 18. Control for baseline differences |
| 19. Confounding: valid and reliable measures, consistently implemented? |
| 20. Confounding, effect modification: important variables were considered? |
| Analysis outcome |
| 21. Loss to follow-up: assessment of impact? |
| 22. Intermediate variables not controlled for? |
| 23. Statistical methods appropriate? |
| Interpretation |
| 24. Results: believable? |
| Reporting |
| 25. Source of funding identified? |
| Added questions |
| 26. Potential for immortal time bias |
| 27. Formulary restrictions present? |
| 28. Confounding by indication present? |
| 29. Unmeasured confounding present? |

Table S5e. RTI Item Bank Quality Assessment Results, Individual Studies

|  |  | Domains and Question Numbers in Each Domain | | | | | | | | | | | | | | | | | | | | | | | | | | | | | | |
| --- | --- | --- | --- | --- | --- | --- | --- | --- | --- | --- | --- | --- | --- | --- | --- | --- | --- | --- | --- | --- | --- | --- | --- | --- | --- | --- | --- | --- | --- | --- | --- | --- |
|  |  | Sample Definition and Selection | | | | | | | Intervention/ Exposure | Outcome | Creation of Treatment Groups | | | Blinding | Soundness of Information | | | Follow–up | | | Analysis Comparability | | | Analysis Outcome | | | Interpretation | Reporting | Added Questions | | | |
| Author | Year | 1a | 1b | 2 | 3 | 4 | 5 | 6 | 7 | 8 | 9 | 10 | 11 | 12 | 13 | 14a | 14b | 15 | 16 | 17 | 18 | 19 | 20 | 21 | 22 | 23 | 24 | 25 | 26 | 27 | 28 | 29 |
| Chou^a^ | 2011 | + | + | – | + | + | • | – | – | + | + | – | – | • | ? | + | + | • | + | ? | – | – | – | – | – | ? | – | + | + | – | – | – |
| Delea | 2003 | + | + | + | + | – | • | + | + | + | + | + | + | • | + | – | + | – | + | ? | + | + | + | – | + | ? | – | + | – | ? | + | + |
| Graham^a^ | 2010 | + | + | + | ? | + | • | + | ? | + | + | + | ? | • | + | + | + | + | – | ? | + | + | + | • | + | + | + | + | + | ? | + | + |
| Habib | 2009 | + | + | + | + | + | • | – | ? | + | + | + | ? | • | + | + | + | + | + | ? | + | + | ? | ? | + | + | + | + | + | – | + | + |
| Hartung | 2005 | + | + | + | + | + | • | – | ? | + | – | + | ? | • | + | + | + | • | + | • | + | + | ? | • | + | + | ? | – | • | ? | – | – |
| Horsdal^a^ | 2008 | + | + | + | + | + | • | + | + | + | + | + | + | • | + | + | + | + | + | ? | + | + | + | – | – | ? | + | + | + | ? | + | + |
| Horsdal | 2009 | + | + | + | ? | + | • | + | + | + | + | – | + | • | + | + | + | + | + | ? | + | + | + | – | – | ? | + | + | + | ? | + | + |
| Hsiao^a^ | 2009 | + | + | + | ? | + | • | – | + | + | + | + | + | • | ? | – | + | • | + | ? | + | + | ? | – | + | ? | – | + | – | ? | + | + |
| Hsiao | 2010 | + | + | + | ? | + | • | + | + | + | + | + | + | • | – | – | + | • | + | ? | + | + | ? | – | + | ? | ? | + | + | ? | + | + |
| Juurlink^a^ | 2009 | + | + | + | ? | + | • | + | ? | + | + | + | + | • | ? | ? | + | • | + | ? | + | + | + | ? | + | + | + | + | + | ? | + | + |
| Karter^a^ | 2005 | + | + | + | + | + | • | + | + | + | + | – | ? | • | + | + | + | • | – | ? | + | + | + | ? | + | + | + | + | – | ? | + | – |
| Koro^a^ | 2005 | + | + | + | + | + | • | + | + | + | + | + | + | • | + | + | + | • | ? | ? | + | + | + | ? | + | + | ? | – | • | ? | – | – |
| Lipscombe | 2007 | + | + | + | + | + | • | + | + | + | + | + | ? | • | + | + | + | • | + | ? | + | + | + | ? | + | + | + | + | • | – | – | + |
| Loebstein^a^ | 2011 | + | + | ? | ? | + | • | + | ? | + | + | + | + | • | + | + | + | • | + | ? | + | – | + | ? | – | + | ? | – | + | – | – | + |
| McAlister^a^ | 2008 | + | + | + | ? | + | • | + | ? | + | + | + | + | • | + | – | + | • | + | ? | + | + | ? | ? | + | + | + | + | + | ? | + | + |
| Rajagopalan | 2004 | + | + | + | ? | + | • | + | ? | + | – | + | + | • | + | – | + | • | + | ? | + | + | + | – | + | ? | – | – | + | ? | – | – |
| Toprani | 2011 | + | + | + | + | + | • | + | + | + | + | + | – | • | + | – | + | • | + | ? | + | ? | ? | ? | – | – | – | – | – | ? | – | – |
| Tzoulaki^a^ | 2009 | + | + | + | + | + | • | + | + | + | + | + | + | • | ? | + | + | + | + | ? | + | + | + | ? | + | + | + | + | + | ? | – | – |
| Wertz^a^ | 2010 | + | + | + | ? | + | • | – | + | + | + | + | + | • | + | + | + | • | + | ? | + | + | + | ? | + | ? | ? | + | + | ? | + | – |
| Winkelmayer^a^ | 2008 | + | + | + | ? | + | • | + | ? | + | + | + | ? | • | + | + | + | • | + | • | + | + | + | + | + | + | + | + | + | + | + | + |

^a^ Included in meta-analysis

Key: • = not applicable ; + = low risk of bias; ­– = high risk of bias; ? = unclear risk of bias.

Figure S1e. Heart Failure Relative Risk (Random Effects) in New Users: Rosiglitazone Compared to Pioglitazone


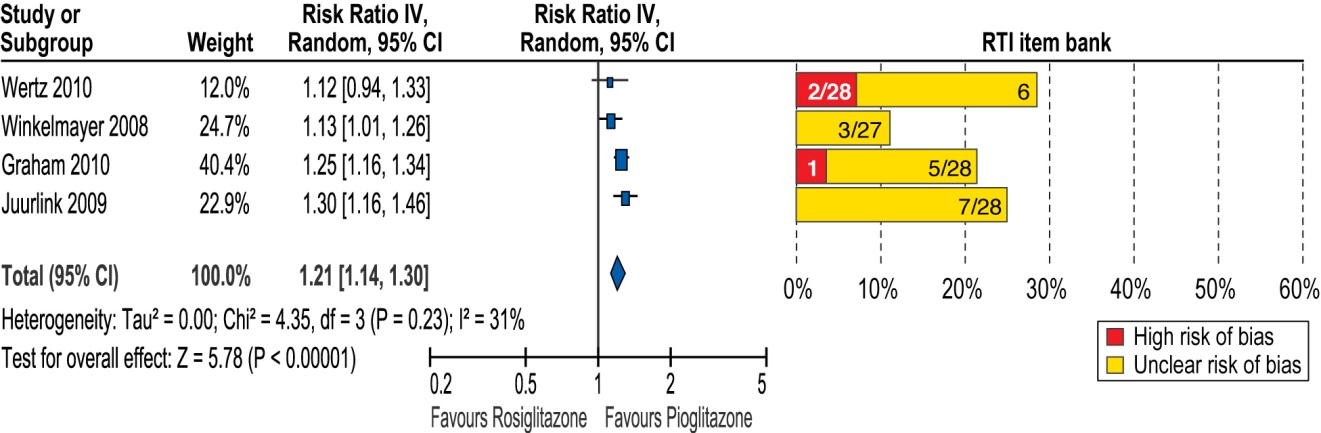


Figure S2e. Heart Failure Relative Risk (Random Effects) in Rosiglitazone Users Compared With Metformin Users, by Type of Regimen


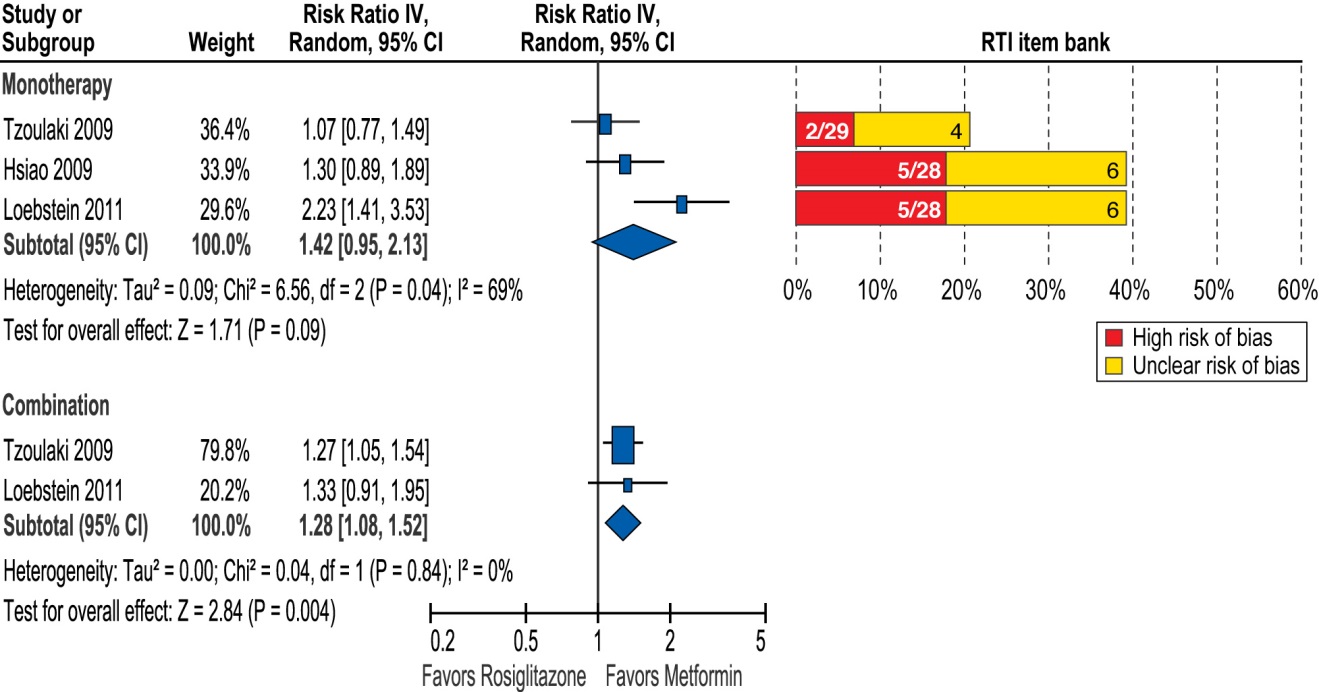


Figure S3e. Heart Failure Relative Risk (Random Effects) in Sulfonylurea Users Compared With Metformin Users, by Type of Use


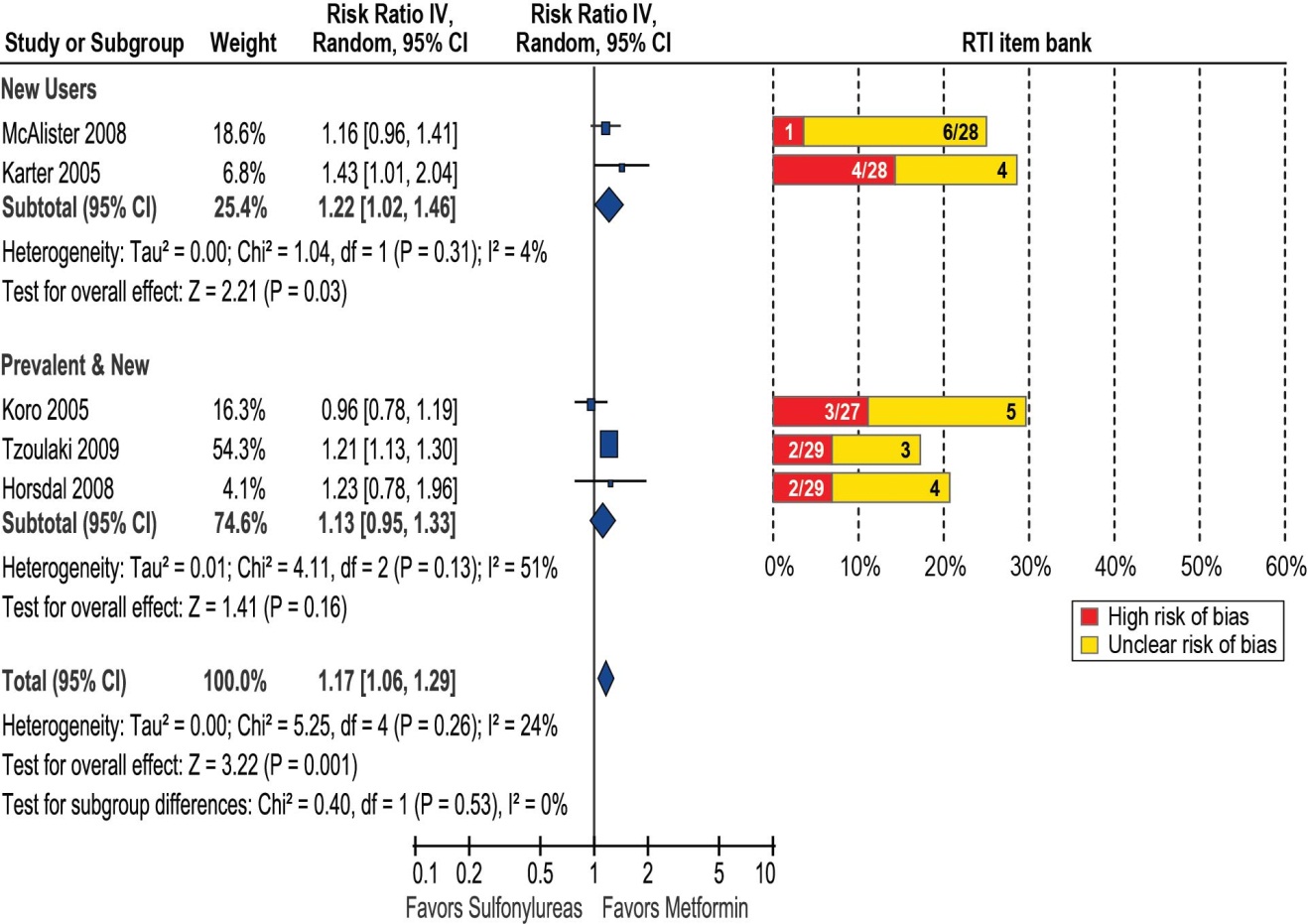


Figure S4e. Funnel Plot: Heart Failure Relative Risk for Rosiglitazone Users Compared With Pioglitazone Users (5 Studies)

Note: Relative risk (RR) is plotted on the horizontal axis and an estimate of its precision, SE (log RR), on the vertical axis.

Figure S5e. Funnel Plot: Heart Failure Relative Risk for Sulfonylureas Users Compared With Metformin Users (5 Studies)

Note: Relative risk (RR) is plotted on the horizontal axis and an estimate of its precision, SE (log RR), on the vertical axis.

MOOSE Checklist

| Criteria | | Brief description of how the criteria were handled in the meta-analysis |
| --- | --- | --- |
| Reporting of background should include | |  |
| √ | Problem definition | Patients with type 2 diabetes mellitus (T2DM) are at high risk of heart failure. A summary of the effects of blood glucose–lowering drugs other than glitazones on the risk of heart failure in routine clinical practice is lacking. |
| √ | Hypothesis statement | The effects of blood glucose–lowering drugs on the risk of heart failure in routine clinical practice, other than the risk observed for glitazones, has not been systematically reviewed and integrated.  We did not have a pre-specified hypothesis – only an interest in filling the noted knowledge gap. |
| √ | Description of study outcomes | Heart failure |
| √ | Type of exposure or intervention used | Non-insulin blood glucose–lowering medications |
| √ | Type of study designs used | Observational studies |
| √ | Study population | Patients with T2DM receiving pharmacologic treatment with non-insulin l blood glucose-lowering medications |
| Reporting of search strategy should include | |  |
| √ | Qualifications of searchers | The credentials of the investigators CVL, MP, NRG, LH and SPG are indicated in the author list. MH, Information Services Specialist, is listed in the acknowledges for his support and contributions. |
| √ | Search strategy, including time period included in the synthesis and keywords | The search period covered until November 2011; an update of the literature search was conducted in June 2014. The search terms for the Medline search are provided with the Additional information.  The search strategy used in other databases is available upon request |
| √ | Databases and registries searched | PubMed, Embase and the Cochrane Library |
| √ | Search software used, name and version, including special features | We did not employ special search software other than PubMed Advanced Search Builder available through Medline. EndNote was used to merge retrieved citations and eliminate duplications |
| √ | Use of hand searching | We hand-searched bibliographies of retrieved papers for additional references. |
| √ | List of citations located and those excluded, including justifications | Details of the literature search process are outlined in the flow chart (Figure 1). The list of located citations is available upon request |
| √ | Method of addressing articles published in languages other than English | There were no language restrictions in the literature search. The few papers we identified that were not written in English had an abstract in English and did not meet inclusion criteria. |
| √ | Method of handling abstracts and unpublished studies | We included only published observational studies, not abstracts or unpublished studies. |
| √ | Description of any contact with authors | Authors of a few studies were contacted with requests for point estimates and confidence intervals when the data to extract appeared only in figures in the original study, or for clarifying information for study inclusion decisions. |
| Reporting of methods should include | |  |
| √ | Description of relevance or appropriateness of studies assembled for assessing the hypothesis to be tested | Detailed inclusion and exclusion criteria were described in the methods section, within the “Study selection and data abstraction” paragraph. |
| √ | Rationale for the selection and coding of data | We extracted information on study population, study design, exposure, outcome and covariates, and results. A summary is shown in Table 1. |
| √ | Assessment of confounding | We extracted results that were adjusted, at a minimum, for age and sex, and preferred the most extensively adjusted results provided in each publication, and extracted details on confounders and confounding adjustment in each included study. We conducted sensitivity analyses based on study design, regimen, type of drug use, type of event and age range of the source population. |
| √ | Assessment of study quality, including blinding of quality assessors; stratification or regression on possible predictors of study results | We assessed the quality of each study included in the systematic review using two tools that investigate the risk of bias in several domains, the Newcastle-Ottawa Quality Assessment Scale (NOS) and the RTI item bank on risk of bias and precision (the latter conducted independently by two assessors blinded to the other assessor´s rating). We did not perform analyses based on quality ratings, but we resorted to them to interpret results. |
| √ | Assessment of heterogeneity | Heterogeneity of the studies was assessed by graphical inspection of forest plots and with Cochran’s *χ^2^* test of homogeneity. *Tau^2^* is also presented as an estimate of the between-study variance. The Higgins *I^2^* statistic was used to describe the percentage of between-study variability in estimates of the total variability that is attributable to true heterogeneity rather than chance. |
| √ | Description of statistical methods in sufficient detail to be replicated | Description of methods of meta-analyses, heterogeneity, subgroup analysis and assessment of publication bias are detailed in the methods. |
| √ | Provision of appropriate tables and graphics | We included tables and figures in the manuscript and in the online materials |
| Reporting of results should include | |  |
| √ | Graph summarizing individual study estimates and overall estimate | Figure 2, Figure 1e, Figure 2e, Figure 3e. |
| √ | Table giving descriptive information for each study included | Table 1 |
| √ | Results of sensitivity testing | Table 2, Table 3 |
| √ | Indication of statistical uncertainty of findings | 95% confidence intervals were presented with all individual and summary estimates, I^2^ values and results of subgroup analyses |
| Reporting of discussion should include | |  |
| √ | Quantitative assessment of bias | We used the Newcastle-Ottawa Quality Assessment Scale (NOS) and the RTI item bank to quantify the risk of bias. |
| √ | Justification for exclusion | No exclusion criteria were implemented |
| √ | Assessment of quality of included studies | We discussed the results of the NOS and RTI item bank, and potential reasons for the observed heterogeneity. |
| Reporting of conclusions should include | |  |
| √ | Consideration of alternative explanations for observed results | Among other, we discussed which potential unmeasured confounders may have caused residual confounding, the potential role of adjusting for intermediate factors that could have underestimated the relative risk, the potential for indication bias or the impact of some quality aspects observed in the individual studies that qualify them as at high risk of bias. |
| √ | Generalization of the conclusions | The overall results of this meta-analysis suggest that patients with T2DM using either glitazones or sulfonylureas might be at greater risk of heart failure than metformin users. However, indication bias might account for part of the differential effect between these medications. |
| √ | Guidelines for future research | We recommend large studies including newer available blood glucose–lowering therapies. When possible, the use of a common reference group is recommended to address the direct comparison of effect estimates. |
| √ | Disclosure of funding source | This research has received funding from the European Community's Seventh Framework Programme (FP7/2007-2013) under grant agreement number 282521—the SAFEGUARD project |
